# Syntactic and Story Structure Complexity in the Narratives of High- and Low-Language Ability Children with Autism Spectrum Disorder

**DOI:** 10.3389/fpsyg.2017.02027

**Published:** 2017-11-20

**Authors:** Eleni Peristeri, Maria Andreou, Ianthi M. Tsimpli

**Affiliations:** ^1^Language Development Lab, Department of English Studies, Aristotle University of Thessaloniki, Thessaloniki, Greece; ^2^Department of English, School of Arts and Humanities, University of Cologne, Cologne, Germany; ^3^Department of Theoretical and Applied Linguistics, University of Cambridge, Cambridge, United Kingdom

**Keywords:** autism, language ability, narratives, sentence complexity, microstructure, macrostructure

## Abstract

Although language impairment is commonly associated with the autism spectrum disorder (ASD), the Diagnostic Statistical Manual no longer includes language impairment as a necessary component of an ASD diagnosis (American Psychiatric Association, 2013). However, children with ASD and no comorbid intellectual disability struggle with some aspects of language whose precise nature is still outstanding. Narratives have been extensively used as a tool to examine lexical and syntactic abilities, as well as pragmatic skills in children with ASD. This study contributes to this literature by investigating the narrative skills of 30 Greek-speaking children with ASD and normal non-verbal IQ, 16 with language skills in the upper end of the normal range (ASD-HL), and 14 in the lower end of the normal range (ASD-LL). The control group consisted of 15 age-matched typically-developing (TD) children. Narrative performance was measured in terms of both microstructural and macrostructural properties. Microstructural properties included lexical and syntactic measures of complexity such as subordinate vs. coordinate clauses and types of subordinate clauses. Macrostructure was measured in terms of the diversity in the use of internal state terms (ISTs) and story structure complexity, i.e., children's ability to produce important units of information that involve the setting, characters, events, and outcomes of the story, as well as the characters' thoughts and feelings. The findings demonstrate that high language ability and syntactic complexity pattern together in ASD children's narrative performance and that language ability compensates for autistic children's pragmatic deficit associated with the production of Theory of Mind-related ISTs. Nevertheless, both groups of children with ASD (high and low language ability) scored lower than the TD controls in the production of Theory of Mind-unrelated ISTs, modifier clauses and story structure complexity.

## Introduction

Although the Diagnostic and Statistical Manual of Mental Disorders, Fifth Edition (DSM-5) has de-emphasized language ability in the diagnosis of Autism Spectrum Disorders (ASD) by removing the criteria of “age-of-onset” and “no history of language delay” for Asperger's syndrome, language impairment is commonly associated with ASD. In fact, language delay is the most frequent cause of initial referral to specialist services for children with ASD (McMahon et al., 2007). Language ability varies considerably among diagnosed individuals, with 30% lacking minimal spoken language despite access to intervention (*minimally verbal children*; Kjelgaard and Tager-Flusberg, 2001; Anderson et al., 2007; Tager-Flusberg and Kasari, 2013), while highly-verbal children with ASD tend to exhibit considerable heterogeneity in their language abilities (e.g., Charman, 2004; Kjellmer et al., 2012). Receptive language is usually lower than expressive language in highly-verbal children with ASD (e.g., Hudry et al., 2010), though it is sometimes anecdotally reported that some school-aged children demonstrate relatively good receptive skills, despite their low expressive skills (Kasari et al., 2013). Within this framework, a distinction is often made between children with ASD who have age-appropriate language skills and those who have a language impairment similar to that found in children with Specific Language Impairment (SLI), but whose vocabulary levels and non-verbal cognition are intact (e.g., Rapin and Dunn, 2003; Tager-Flusberg and Joseph, 2003; Tager-Flusberg, 2006). For instance, Tek et al.'s (2014) longitudinal study revealed similar language growth patterns of children with ASD and good language skills with their typically-developing (TD) age-matched peers in a variety of language measures, including grammatical morphemes, vocabulary and sentence complexity, in contrast to an age-matched group with ASD and low verbal skills that exhibited developmental delays across the same language areas.

The long-recognized variation in language ability in ASD suggests that the autistic language phenotype can be partly, yet consistently dissected on the basis of the children's verbal skills. Recently, this assumption has been formalized in the study by Wittke et al. (2017) that designated a specific, grammatically-impaired subgroup of SLI in a large ASD sample. This group performed in the normal range on non-verbal IQ and vocabulary while still showing a specific deficit in grammatical skills, in contrast with a group of language-impaired children with ASD who had significantly below average non-verbal IQ and overall deficits in vocabulary and grammar. Moreover, some grammatical errors were more frequent in the grammatically-impaired group than the group with global language impairment. Consequently, Wittke et al.'s (2017) study has highlighted a structural deficit in a subgroup of children with ASD that was due neither to low non-verbal skills nor to the severity of ASD symptoms, as previously suggested (Harper-Hill et al., 2013). Wittke et al.'s (2017) study focused exclusively on children's grammatical impairment at the single-morpheme level [by reference to Brown's (1973) 14 grammatical morphemes]. To the best of our knowledge, no study has so far investigated the effect of language ability on different aspects of narrative production in children with ASD. Thus, which narrative functions are affected in children with ASD who vary in their language ability but have average non-verbal and verbal IQ, remains unexplored. In the present study, we recruited two groups of children with ASD and normal non-verbal IQ whose language ability, reflected in verbal IQ and expressive vocabulary, was within the normal range, yet, on extreme ends of the scales (high vs. low). We focus on the question of whether such disparity affects narrative production at the microstructural and macrostructural levels of analysis.

The field has now recognized that the variation in the language abilities of children with ASD is partly related to syntactic skills. Narratives have been successfully used in studies with children with ASD to elucidate differences which are not apparent or clearly defined using standardized tools alone. The narratives of children within the spectrum have been shown to include syntactically less complex sentences, omitted morphemes and increased rates of coordination (e.g., Roberts et al., 2004; Eigsti et al., 2007; Marinis et al., 2013; Norbury et al., 2014). However, whether language ability in ASD can affect the production of specific types of subordinate clauses in narratives remains largely unknown. Moreover, most previous studies focus on English-speaking children with ASD. Other languages, in which morphosyntactic features of subordination are richer than in English, have barely been studied. Furthermore, the contribution of pragmatics in the production of some types of subordinate clauses, such as adverbial or relative clauses, has not been specifically addressed in the examination of narratives produced by individuals with ASD. For example, adverbial clauses provide cues to establish coherence relations between the events of a story. As such, these clauses may be particularly challenging for children with ASD due to the pragmatic deficit that defines autism (e.g., Naigles and Chin, 2015; de Marchena and Eigsti, 2016). One of the aims of the present study is to examine the extent to which variation in language ability in children with ASD affects the use of modifier clauses, i.e., adverbial and relative clauses (Hughes et al., 1997). In this respect, the compensatory role of good language ability in planning and producing a coherent and complete plotline for the story is also examined. Children with ASD have been shown to encode the characters' emotions and thoughts, referred to as +Theory of Mind-related Internal State Terms (ISTs) (henceforth, +ToM-related ISTs) less often than TD children (e.g., Siller et al., 2014). The link between subordination and performance in ToM tasks has been commonly found in children with ASD. For instance, Tager-Flusberg (2000) reported that children with ASD experienced greater difficulty than age-matched intellectually-impaired children in extracting the embedded clause of communication verbs in wh-questions (e.g., Why did Bobby say Dad put the cake away?). Moreover, performance on these questions was a strong predictor of children's false belief reasoning abilities. Nevertheless, the effect of high- vs. low-verbal skills in children with ASD on the use of ±ToM-related ISTs and on story structure complexity has not been examined as yet.

The present study aims to fill this gap to contribute to the question of whether good language abilities can compensate for the pragmatic deficit in and its effects on microstructural and macrostructural aspects of narratives. Specifically, all the children with that participated in the present study had non-verbal IQ scores within the normal range of the Wechsler Intelligence Scale for Children (Wechsler, 1992; WISC-III; Greek adaptation and standardization by Georgas et al., 2003). Variation in the group was defined through language ability which was measured by a standardized expressive vocabulary test and the verbal IQ tests of WISC-III (Wechsler, 1992). We measured syntactic complexity by calculating the number of complex sentences in each child' narrative. Complex sentences are those which include more than one clause which can be coordinated or subordinated to the matrix clause. The language of the experimental and the control group is Greek, a language with richer morpho-syntactic distinctions in subordinate clauses than English. Two types of subordinate clauses are examined in narrative microstructure: (i) verb-complement clauses, i.e., clauses selected as complements of a verb in the higher clause, and (ii) modifier clauses, including temporal or causal adverbial clauses and relative clauses modifying subject or object noun phrases in the sentence. One crucial difference between complement and modifier clauses is that complement clauses are selected by the verb, hence their use presupposes lexical and syntactic knowledge (Grimshaw, 1979; Noonan, 1985; Haegeman, 2006). Modifier clauses, on the other hand, are not selected. Instead, they are used for semantic and pragmatic purposes, such as to establish cohesion between events in a narrative by providing causal or temporal information, or by elaborating on the referentiality of the noun phrase (Fox and Thompson, 1990; Vieu et al., 2005). In essence, the use of modifier clauses presupposes morpho-syntactic but also pragmatic skills which guide the conceptual structure and planning of the propositions encoded in the narrative. By distinguishing between complement and modifier subordinate clauses in the narratives of children with we can examine the relative contribution of language as opposed to pragmatics in and the compensatory role of language on pragmatics. The examination of narrative macrostructure, as instantiated in the use of ±ToM-related ISTs and in the complexity of story structure contributes to the same question: higher use of ±ToM-related ISTs and/or story structure complexity for the group of children with and high language skills as opposed to those with low language skills would speak in favor of the compensatory role for language in pragmatics. In contrast, if the two groups of children perform similarly in those macrostructure measures as well as in the use of subordination, it would be concluded that the pragmatic deficit cannot be masked or overcome by good language skills.

### Grammar in ASD: evidence from narrative production

Research in the use of grammar in autism has often relied upon the analysis of children's narratives. Narrative production shows differences among participants with ASD, which depend on language ability: less verbally-able participants tend to produce shorter and syntactically simpler utterances than language-matched controls (Tager-Flusberg, 1995; Tager-Flusberg and Sullivan, 1995; Capps et al., 2000). Findings are less consistent when children with ASD are compared to age-matched TD participants. A number of studies (Losh and Capps, 2003; Diehl et al., 2006; Novogrodsky, 2013; Norbury et al., 2014) found no differences between individuals with ASD and their TD peers with similar language abilities; however, Stirling et al. (2017) did report that children with ASD lagged behind TD children in syntactic complexity in their written narratives. Bishop (2003) also reports that children with ASD aged between 6 and 10 years who had typical non-verbal abilities but low scores on expressive and/or receptive language measures, produced fewer complex sentences than their TD peers. Although, language ability operationalized in terms of expressive and/or receptive language abilities has been used as a qualifier of ASD children's narrative performance (e.g., Norbury et al., 2014; Suh et al., 2014), not much is known about the different types of subordination which may be particularly challenging for children with autism. The current study examines whether different levels of language skills in children with ASD differentially affect the use of complement vs. modifier clauses.

Apart from investigating the microstructure of narratives, i.e., lexical diversity and morpho-syntax, research on narratives and autism has focused on the analysis of macrostructure. This level includes the linguistic encoding of the characters' affective and cognitive states, as well as the encoding of reference which requires appropriate pronominal form-function mappings as the discourse unfolds. An effective narrator not only has to structure the story in an intelligible way so that the listener understands the setting, characters, events, and outcomes of the story (Rumpf et al., 2012), but also needs to identify the motivations and reactions of the characters that embed socially-oriented goals (Stein and Glenn, 1979). The specific domain has been key to systematically revealing the pragmatic deficit in ASD. Pragmatic difficulties have been considered the hallmark of ASD and a domain in which all children within the spectrum, even those with age-appropriate structural language abilities and intelligence, struggle to master to various degrees (Rapin and Dunn, 1997; Landa, 2000; Bishop and Baird, 2001; Kjelgaard and Tager-Flusberg, 2001; Tager-Flusberg et al., 2005; Stefanatos and Baron, 2011). Children with ASD have been shown to produce narratives with more ambiguous referencing (Loveland et al., 1990; Tager-Flusberg, 1995; Manolitsi and Botting, 2011; Novogrodsky, 2013; Norbury et al., 2014; Suh et al., 2014), fewer dialogic interactions among the story characters (Stirling et al., 2017), fewer ISTs referring to the story characters' emotions (e.g., Siller et al., 2014), and inappropriate use of language within context (e.g., Losh and Capps, 2003; Collet-Klingenberg and Franzone, 2008) compared to TD children.

Prior work on language ability in ASD has often focused on its compensatory role in children's ToM deficit. Much of the relevant work has tested prosody and scalar implicatures in sentential contexts (McCann et al., 2007; Pijnacker et al., 2009). Moreover, certain language skills but also abstract thinking have been proposed as factors contributing to success in ToM tasks (e.g., Eisenmajer and Prior, 1991; Happé, 1994; Steele et al., 2003; Milligan et al., 2007; Durrleman et al., 2016). Crucially, ASD children's use of affective terms in narratives has been found to correlate significantly with their performance on ToM tasks, i.e., tasks that tapped into the children's ability to represent their own or other people's mental states, like pretend-play (Blanc et al., 2005), and false belief (Baron-Cohen et al., 1985). Although the causality of the link between ToM and language in autism (or typical language development) is not defined as yet, findings suggest that ASD children's failure to consider the perspectives of others has consequences on the use of language expressing mental states. Thus, affective terms are used considerably less frequently by children with ASD during storytelling compared to TD controls despite ASD children's intact meta-representational skills (Baron-Cohen et al., 1985; Baron-Cohen, 1989). The present study aims to investigate the extent to which the language ability of children with ASD, whose verbal skills are within the normal range but differs in being high and low, may compensate for the ToM deficit in narratives.

### Subordinate clauses in Greek

As already mentioned, one of the aims of the present study is to investigate whether microstructure in ASD, and in particular syntactic complexity in narrative production differs in children whose language abilities lie at the higher and lower end of the normal range. Subordination, and in particular, complement and modifier subordinate clauses are also examined.

Complement clauses are further distinguished in terms of the complementizers which introduce them. Non-interrogative complement clauses in Greek can be introduced by the complementizers *na, oti/pos*, and *pu* [examples (1)–(3) below]. Whereas, in English the distinction in complementation is between finite and non-finite complement clauses which can be introduced with an overt or zero complementizer (e.g., “I know *that*/Ø he left,” “I want Ø to leave”), in Greek the use of a complementizer is obligatory. Morphological distinctions in complementizers are based on Mood (subjunctive *na* vs. indicative *oti/pos*), and factivity (factive *pu* vs. non-factive *oti/pos*). The complementizer *na* is a Mood marker introducing subjunctive clauses (Holton et al., 1997), which are the closest translational equivalents to infinitival clauses in English (Agouraki, 1990; Tsimpli, 1990; Roussou, 1994; Giannakidou, 2009). The complementizer *oti* introduces indicative clauses, while the complementizer *pu* is used to introduce complements of psychological predicates like *lipame* “be-sad,” *metanjono* “regret,” *herome* “be-glad” (Christidis, 1986; Varlokosta, 1994). In terms of differences in the feature complexity of these three complementizers, the subjunctive one is the least complex with respect to finiteness features as it is the only complementizer in Greek which can introduce clauses with underspecified Tense features. Notably, *na*-clauses are the earliest to develop in typically-developing Greek children; the form that the verbs have in *na*-clauses are among the earliest forms used by Greek-speaking children even in matrix contexts (Varlokosta, 1994; Tsimpli, 2005). The present study examines the frequency of use of indicative *oti*/*pos* and factive *pu* vs. the subjunctive *na* complementizers in the children's narratives.

(1) Thelo **na** figho/fighi i Maria.
  want_1SG_ to leave_PRF.NONPST.1SG/3.SG_ the Mary
  “I want (Mary) to leave.”
(2) O Yanis kseri/pistevi **oti/pos** o Kostas apetihe stis eksetasis.
  the Yanis know/believe_3SG_ that the Kostas failed_3SG_ in
  the exams
  “John knows/believes that Kostas failed in
  the exams.”
(3) I Maria lipate **pu** o Kostas apetihe stis eksetasis.
  the Maria is-sad/sorry_3SG_ that the Kostas failed_3SG_ in
  the exams
  “Mary is-sad/sorry that Kostas failed in
  the exams.”

In addition to complement clauses, we examine adverbial and relative clauses which modify an event and a noun phrase, respectively. Adverbial and relative clauses are not selected and therefore optional (Haegeman, 1994). The function and the positioning of adverbial clauses have been argued to be mainly motivated by their pragmatic function. Adverbial clauses usually provide temporal or causal information which modifies the event of the main clause (Haegeman, 2006, 2010) [examples (4)–(5) below]. In connected speech, adverbial clauses establish cohesive links between the events of a story, thus contributing to the complexity of narration (Shapiro and Hudson, 1991; Andreou, 2015). Moreover, they enrich the propositional content of the complex sentence in which they occur, and assume organizational functions at the pragmatics level (Bestgen and Vonk, 2000; Vieu et al., 2005). Relative clauses [exemplified in (6) below] are embedded within nominal phrases and are thought to function like predicates or modifiers of a head noun (e.g., in “The man that I saw,” the relative clause “that I saw” modifies the noun phrase “the man”).

(4) O babas mu efije **otan** imun 7
  the dad my leave_PRF.NONPST.3SG_ when was_1SG_ 7.
  “My dad left when I was 7.”
(5) I mathitria lipithike **epidhi** to ajori tin enohlise
  the student got-sad_NONPAST.3SG_ because the boy her_CL.3P.SG_
  irritated_NONPAST.3SG_
  “The student got sad because John irritated her.”
(6) To vivlio **pu** ajorasa itan skismeno.
  the book that bought_PAST.1SG_ was torn
  “The book that I bought was torn.”

According to corpus-studies (Fox and Thompson, 1990; Biber et al., 1998), the discourse function of relative clauses is to ground the head entity with respect to discourse information and to elaborate further on its referential properties. The modifier status of both adverbial and relative clauses allows us to group them together under the modifier clause category. Although all subordinate clauses presuppose lexical and morphosyntactic skills (microstructural properties), modifier clauses also build on pragmatic organization skills. The distinction drawn between complement and modifier clauses receives support from studies on monolingual typical development, showing that complement clauses emerge earlier and the timing difference between complement and modifier clauses is considerable (Diessel and Tomasello, 2001; Diessel, 2009).

## The current study

This study builds on previous research examining the narrative performance of children with ASD (e.g., Perner et al., 1987; Wellman and Woolley, 1990; Lewis et al., 1994; Sullivan et al., 1994; Tager-Flusberg, 1995; Novogrodsky, 2013; Norbury et al., 2014; Siller et al., 2014; Stirling et al., 2017) and addresses the role of language ability on performance in narrative microstructure and macrostructure. In order to capture variability in the language profiles of the children with ASD, we used standardized measures of both verbal IQ (VIQ) based on the children's performance in the verbal scales of the Greek version of WISC-III (Wechsler, 1992), and expressive vocabulary (Vogindroukas et al., 2009); adaptation from Renfrew (1997). This targeted recruitment allowed us to identify two subgroups of children in the spectrum, those with expressive vocabulary and verbal IQ scores in the higher end of the normal scale (henceforth, ASD-HL), and those in the lower end of the normal scale (henceforth, ASD-LL).

The study's research questions and hypotheses are the following:

***Question 1***. Will the difference in the language ability of the two groups of children with ASD affect frequency of use of complex (i.e., coordinate and subordinate) clauses?
***Hypothesis 1***. Based on previous research (e.g., Tager-Flusberg, 1995; Tager-Flusberg and Sullivan, 1995; Capps et al., 2000; Kjelgaard and Tager-Flusberg, 2001; Bishop, 2003; Eigsti et al., 2007) showing that children with ASD and concomitant language impairment produce fewer syntactically complex sentences compared to TD children, we expect that the ASD-LL group will exhibit fewer syntactically complex sentences than ASD-HL and TD children.
***Question 2***. Will the difference in the language ability of the two groups of children with ASD affect frequency of use of the different types of complementizers?
***Hypothesis 2***. We hypothesize that differences in language ability will affect the diversity of complementizers. Specifically, we expect *oti*-(“that”) and *pu*-(factive “that”) complements to be particularly compromised for the ASD-LL group only, due to the fact that these complementizers select verb forms which are fully-specified for Tense and Aspect in contrast to *na*-(subjunctive) complements which are temporally underspecified (Agouraki, 1990; Tsimpli, 1990; Roussou, 1994). We do not expect differences to emerge between ASD-HL and TD children in the use of *oti*-(“that”) and *pu*-(factive “that”) clauses due to the fact that the former group of children is predicted to compensate for the computational demands of complementation by means of their high language ability.
***Question 3***. Will children with ASD and higher language skills perform better than their ASD-LL peers in the use of adverbial and relative clauses?
***Hypothesis 3***. Assuming that the production of adverbial and relative clauses draws more heavily on discourse and pragmatics relative to verb-complement clauses (Grimshaw, 1979; Fox and Thompson, 1990; Vieu et al., 2005), we predict lower frequency of use of adverbial and relative clauses for both groups of children with ASD relative to TD children. If, on the other hand, ASD-HL children recruit high language skills as a compensatory mechanism for their pragmatic deficit during retelling, we expect this group to produce higher rates of modifier clauses than their ASD-LL peers and similar rates to their TD peers.
***Question 4***. Will children with ASD and higher language skills perform better than ASD-LL children in the use of ±ToM-related ISTs?
***Hypothesis 4***. Based on previous research (De Villiers, 2000; Tager-Flusberg and Joseph, 2005; Schick et al., 2007; Lind and Bowler, 2009; De Villiers and De Villiers, 2014; Durrleman and Franck, 2015; Durrleman et al., 2016) showing that children with autism tend to over-rely on the structural representation of complement clauses to compensate for their mentalizing deficit, we expect ASD-HL children to produce higher rates of +ToM-related ISTs compared to the ASD-LL group and similar rates to their TD peers supported by their good language skills. On the other hand, since this compensatory process is not essential to non-mentalizing expressions, such as −ToM-related ISTs, both groups of children with ASD are expected to score lower than TD children in this category.
***Question 5***. Will children with ASD and higher language skills perform better than their ASD-LL peers in the macrostructure measure of story structure complexity?
***Hypothesis 5***. Previous research shows that children with ASD have difficulty structuring narratives in a coherent manner irrespective of age and non-verbal abilities (Loveland et al., 1990; Losh and Capps, 2003; Diehl et al., 2006; Collet-Klingenberg and Franzone, 2008; Stirling et al., 2017). If encoding story structure draws on the child's language skills, then the ASD-HL is expected to outperform the ASD-LL group and perform similarly to the TD group. On the other hand, if retelling a story is more likely to tap into discourse management skills and world knowledge instead of formal language skills alone, we predict that high language ability will have a minimal effect on story structure complexity across the two groups of children with ASD. If this holds true, both groups of children with ASD are expected to perform lower than TD children in this measure.

Finally, in order to explore possible interactions between microstructural and macrostructural measures in the children's narratives, partial correlation analyses between the specific variables are carried out, after controlling for the children's verbal IQ and expressive vocabulary scores.

## Materials and methods

### Participants

A sample of 30 monolingual Greek-speaking children with ASD [mean age: 9.2 yrs. (*SD*: 1.9), age range: 6.1–12.4, all male] was tested. The children were recruited from mainstream state primary schools' inclusion classrooms. They all met criteria for ASD based on expert clinical judgment of the child's social-adaptive functioning conducted by a child psychiatrist, which was confirmed by the Autism Diagnostic Interview-Revised (Lord et al., 1994). Children were assessed with the VIQ and performance IQ (PIQ) scales of the Greek version of WISC-III (Wechsler, 1992; Greek adaptation and standardization by Georgas et al., 2003). All the children had a PIQ score of 83 or above [mean: 108.9 (*SD*: 15.3), range: 83–142]. Language ability was additionally tested with an expressive vocabulary test (Vogindroukas et al., 2009) standardized for 3–10 year old Greek-speaking monolingual children. This word-finding task includes 50 pictures depicting commonplace objects which each child was required to name. Testing was discontinued if the child failed to respond correctly in five consecutive trials. Each correct naming was given one point, so that the maximum score was 50.

The VIQ score of the Weschler Intelligence Scale for Children has been used in prior studies as an indicator of the level of impairment in ASD children's language functioning (Lincoln et al., 1988, 1995; Happé, 1994; Bavin et al., 2014), but it has been insufficient for addressing variability on its own (e.g., Dawson et al., 2007; Nader et al., 2016). As such, expressive vocabulary was also used as a screening measure to characterize ASD children's language profile.

In line with a number of previous studies (e.g., Semel et al., 1987; Marton and Schwartz, 2003; Reilly et al., 2004; Falcaro et al., 2007; Norbury et al., 2014, among many others), children with language scores 1.5 or more standard deviation (*SD*) below the mean were considered as having low language ability. The VIQ score of the rest of the children with ASD was very close to that of the TD children (see Table 1). By using a cut-off of 81 in VIQ (i.e., 1.5 *SD* below the VIQ mean of all the children with ASD) and a cut-off of 33 in the expressive vocabulary task (i.e., at least 1.5 *SD* below the mean expressive vocabulary score of all the children with ASD), the children with ASD formed a high- and a low-language ability group: 16 high-language ability children with ASD (ASD-HL; mean age: 9.2 (*SD*: 1.8), age range: 6.7–12.4) and 14 low-language ability children with ASD (ASD-LL; mean age: 9.1 (*SD*: 2.1), age range: 6.1–12.0). The children with ASD were age-matched with 15 TD monolingual Greek-speaking children (TD; mean age: 9.3 yrs. (*SD*: 1.7), age range: 7.3–12.0). The TD children were selected so that they had normal hearing, no speech, emotional, or behavior problems, as well as no observed neurological, articulation, and phonological deficits.

**Table 1:** Number of children, age, expressive vocabulary, verbal IQ, and performance IQ by Group.

**Group**	**Age**	**Expressive Vocabulary**	**Verbal IQ**	**Performance IQ**
	**M (*SD*) range**	**M (*SD*) range**	**M (*SD*) range**	**M (*SD*) range**
ASD-HL (*N* = 16)	9.2 (1.8) 6.7–12.4	42.9 (2.5) 39–47	113.6 (3.3) 108–119	107.8 (10.3) 83–142
ASD-LL (*N* = 14)	9.1 (2.2) 6.1–12.0	32.7 (2.6) 29–38	79 (2.1) 77–81	110.1 (10.7) 90–142
TD (*N* = 15)	9.3 (1.7) 7.1–12	42.3 (3.6) 35–50	111.1 (8.1) 99–127	112.5 (10.1) 99–134

*ASD-HL, high-language children with Autism Spectrum Disorder; ASD-LL, low-language children with Autism Spectrum Disorder; TD, typically-developing children; M, mean; SD, standard deviation*.

The parents of the children gave written informed consent in accordance with the Declaration of Helsinki, and anonymity of the children and their families was protected. The study with the TD children was carried out in accordance with the recommendations in the *Guide for Research Study Approval* of the Greek Institute for Educational Policy. The parents of the children with ASD gave written consent on the administration of the tasks and on the dissemination of the results for research purposes in strict accordance with the recommendations in the *Guide for the Differential Diagnosis and Intervention for Children with Special Educational Needs* of the Greek Ministry of Education.

A one-way ANOVA analysis with age as the dependent variable indicated that there were no significant differences across groups in age, *F*_(2, 44)_ = 0.75, *p* = 0.928. The three groups differed significantly in both their expressive vocabulary ability, *F*_(2, 44)_ = 25.559, *p* < 0.001, η^2^ = 0.238, and VIQ scores, *F*_(2, 44)_ = 206.728, *p* < 0.001, η^2^ = 0.855. Subsequent *post-hoc* Bonferroni tests showed that the ASD-LL group scored significantly lower in expressive vocabulary and in VIQ than both the ASD-HL and TD children (*p* < 0.001 for all differences). There was no significant difference between the ASD-HL and the TD group in either expressive vocabulary (*p* = 0.919) or VIQ scores (*p* = 0.371) (see Table 1). Furthermore, the three groups did not differ in their performance IQ scores [*F*_(2, 44)_ = 0.428, *p* = 0.654, η^2^ = 0.141].

### Narrative production task

#### Materials

Children's oral retellings were elicited by using a single picture story from the Edmonton Narrative Norms Instrument (ENNI; Schneider et al., 2005) that has been designed to collect narrative data from children aged 4–9 through storytelling. The story used in the present study was the A3 *Giraffe/Elephant* story, which includes eight pictures and consists of three complete episodes (see Appendix A for the pictures that provided the prompts for the narrative, and Appendix B for the original story that the children had to retell).

A minimum of 15 verb clauses was a prerequisite for including a child' narrative in our sample. Moreover, to see if the three groups were comparable in terms of the length of their narratives we ran a one way-ANOVA analysis, with the results showing that there is no group effect in narrative length, *F*_(2, 44)_ = 1.001, *p* = 0.376, η^2^ = 0.213, which was measured in verb clauses (ASD-HL: 25 (*SD*: 4.8); ASD-LL: 22.9 (*SD*: 5.1); TD: 25.5 (*SD*: 5.6). Furthermore, following relevant literature in the field (Tweedie and Baayen, 1998; McCarthy, 2005) we used square root in order to assure that the narratives produced by the children could be compared.

#### Procedure

Each child was tested individually at a location most convenient for the child's parents (i.e., either at the child's home or at a private diagnostic center). The child listened to the story through headphones on the computer screen while viewing two pictures (and a single picture once) per slide. While the child listened to the story, a female adult unfamiliar with the purposes of the study was present in the room. Once the story finished, the child viewed all 13 pictures on a single slide on the computer screen and was asked to retell the story to the examiner, who entered the room only after the child had listened to the whole story and sat opposite the child not being able to see the pictures on the screen.

#### Transcription and scoring of narratives

Children's retellings were audiotaped and transcribed by the first author. One fourth of this sample (25%) was randomly selected and re-transcribed by the second author. Transcripts were then compared word-for-word, with the comparison reaching 99% agreement. Examples of the transcripts of the narratives of a TD, an ASD-HL, and an ASD-LL child are cited in Appendix C).

Both microstructural and macrostructural properties of each child's narrative transcript were scored manually. For microstructure, the scores for the following linguistic categories were calculated: (1) lexical diversity, i.e., number of different types of content words divided by the total number of content-word tokens; (2) syntactic complexity, i.e., number of complex (i.e., coordinate and subordinate) sentences divided by the total number of simple and complex sentences; (3) subordination index, i.e., number of subordinate clauses divided by the total number of complex sentences; and, (4) types of subordination, which include total counts of verb-complement clauses, and modifier, i.e., adverbial and relative clauses in each child's narrative. Complement clauses were further split into two different categories based on the type of the complementizer used to introduce them, i.e., subjunctive *na* complement and indicative *oti*-(that)/*pu*-(that-factive) complement clauses (Mastropavlou and Tsimpli, 2011). Due to the fact that the ASD-LL group produced no *pu*-(that-factive) complement clauses, while ASD-HL children produced very few instances of *pu*-clauses (Mean: 0.8) in their narratives, we opted to merge *oti*-(that) and *pu*-(that-factive) complement clauses and analyze them as a single category due to their shared requirement for Tense and Aspect specification. Examples of types of complement clauses (see examples 7–9) produced by the children with ASD in their narratives are cited below:

Complement clauses:

(7) **na**-complement.
  Theli **na** vutisi mesa stin pisina.
  want_NONPAST.3SG._ to dive_SUBJ.PAST.3SG._ in the swimming pool
  “(She) wants to dive in the swimming pool.”
(8) **oti**-complement.
  Idhe **oti** to aeroplanaki epese stin pisina.
  saw_PAST.3SG_ that the aeroplane fell_PAST.3SG._ in the
  swimming pool.
  “(She) saw that the aeroplane fell in the
  swimming pool.”.
(9) **pu**-complement.
  Harike **pu** pire to aeroplanaki.
  was-happy_PAST.3SG._ that took_PAST.3SG._ the aeroplane
  “(She) was happy that she took back the aeroplane.”

For macrostructure, the following scores were calculated: (1) diversity of +ToM-related ISTs, i.e., number of unique lexical items expressing positive or negative emotion (e.g., *sad, angry, happy*) and mental verbs (such as *think, wonder*) divided by the total count of +ToM-related tokens; (2) diversity of -ToM-related ISTs, i.e., number of unique perceptual (such as *see, hear*), physiological (such as *thirsty, hungry*), and communication (such as *shout, say*) terms divided by the total count of -ToM-related tokens (Gagarina et al., 2012; Tsimpli et al., 2016). The third macrostructural measure included in the analyses was that of story structure complexity (Story Grammar Model; Stein and Glenn, 1979). Each of the three episodes of the story consisted of a Goal of a main character (MC), an Attempt that the MC makes to reach the goal, and the Outcome of the MC's Attempt. The child was awarded three points in each episode for the correct production of Goal, Attempt and Outcome, two points for producing two elements, the Outcome being required in combination with the Goal or the Attempt, one point for producing Goal and Attempt only, and zero points for expressing only one element. Finally, two points were also awarded for the correct reproduction of the place and the time (i.e., the Setting), and one point for introducing the four characters of the story. The maximum score for story structure complexity was 15.

The analysis of both microstructural and macrostructural variables was conducted by the first author and interrater reliability checks were conducted by the second author on 15 (33%) out of the 45 coded transcripts, selected randomly with equal representation of diagnostic (ASD vs. TD) and language ability level (ASD-HL vs. ASD-LL) criteria. Inter-rater reliability was 95.8%, and all discrepancies were resolved through discussion.

## Results

### Group comparisons: microstructural variables

Table 2 presents the raw data (i.e., total counts) for the microstructural variables. Specifically, we present lexical diversity and numbers of simple and complex (coordinate, subordinate) clauses, the syntactic complexity and the subordination index, the number of *na*- (subjunctive) and *oti*-(that)/*pu*-(that-factive) complementizers, and, the numbers of verb-complement and modifier, i.e., adverbial and relative clauses, for each of the three groups. Comparisons among the three groups were analyzed using the Chi-Squared test. In addition, the data were examined by estimating a Bayes factor (BF) using Bayesian Information Criteria (Wagenmakers, 2007). This compares the fit of the data under the null hypothesis compared to the alternative hypothesis, so that a BF < 1 implies substantial evidence for the null hypothesis, according to which there are no group differences in the dependent variable tested, and BF > 1 implies substantial evidence for the alternative hypothesis, which states that there are differences in group performance.

**Table 2:** Group means (and *SD*s) of total counts and proportions (%) for microstructural measures.

**Microstructural measure**	**ENNI (A3) story**	**ASD-HL (*N* = 16)**	**ASD-LL (*N* = 14)**	**TD (*N* = 15)**	**Chi-square (χ^2^)-value**	**Bayesian statistics (BF)**	Interpretation ^*^p < 0.05, ^**^p < 0.005, ^***^p < 0.001
Lexical diversity (%)	65.1	60.5 (7.7)	57.7 (7.1)	56.6 (10.2)	1.69	0.044	ASD-HL = ASD-LL = TD
Simple clause	15 (34.1%)	7.8 (4.1)	8.3 (2.6)	6.1 (2.5)	2.30	0.067	ASD-HL = ASD-LL = TD
Coordinate Clauses	8 (18.2%)	13.2 (5.5)	8 (2.0)	9.3 (5.0)	5.14	8.428	ASD-HL > ASD-LL^**^ ASD-HL > TD^*^
Subordinate Clauses	25 (56.8%)	7.8 (3.8)	5.1 (4.3)	11.1 (2.8)	4.32	7.648	ASD-LL < TD^**^ ASD-HL < TD^*^ ASD-LL = ASD-HL
Syntactic complexity (%)	68.7	72.6 (13.5)	60.1 (12.2)	76.8 (7.4)	19.76	5.039	ASD-LL < TD* ASD-HL = ASD-LL ASD-HL = TD
Subordination index (%)	75.7	37.1 (14.1)	35.1 (16.4)	56.3 (13.1)	9.38	6.389	ASD-HL, ASD-LL < TD^*^ ASD-HL = ASD-LL
*na*- (subjunctive) complementizer	10 (22.7%)	0.37 (1.1)	3.2 (1.8)	0.6 (0.7)	7.01	0.664	ASD-HL = ASD-LL = TD
*oti*-(that)/*pu*-(that-factive) complementizer	5 (11.4%)	5.6 (2.3)	0.6 (1.4)	5.6 (2.3)	7.32	12.360	ASD-LL < ASD-HL, TD^**^ ASD-HL = TD
Verb-complement clauses	15 (34.1%)	6 (2.3)	3.7 (2.6)	6.2 (2.5)	24.99	1.650	ASD-LL < ASD-HL, TD^*^ ASD-HL = TD
Modifier clauses	7 (15.9%)	2.5 (3.1)	1.5 (2.8)	5 (1.5)	38.52	13.49	ASD-HL < TD^*^ ASD-LL < TD^**^ ASD-HL = ASD-LL

*ASD-HL, high-language children with Autism Spectrum Disorder; ASD-LL, low-language children with Autism Spectrum Disorder; TD, typically-developing children; ENNI: Edmonton Narrative Norms Instrument (Schneider et al., 2005)*.

In addition to the presentation of the data from the three experimental groups, all the Tables provide information on the microstructure and macrostructure of the ENNI (A3) story that the children listened to and were asked to retell. To explore the question whether the narrative output of the children differed from the original story on microstructure and macrostructure, we undertook a series of qualitative comparisons targeting the specific narrative properties; to this end, and for each dependent variable, we used proportions by dividing each child's raw scores by the total number of verb clauses. This approach allowed us to detect specific micro- and macrostructural domains in which ASD-HL or/and ASD-LL, as well as TD children's performance deviated substantially from the original story which was used as the baseline. The data is reported in section Comparisons between Group Scores and the Original Story, **Table 4** below.

χ^2^ analyses showed that the groups differed significantly on the number of coordinate and subordinate clauses (χ^2^ > 4.32, *p*s < 0.05), as well as on the syntactic complexity and subordination indices (χ^2^ > 9.38, *p*s < 0.005). Further chi-square analyses revealed that the ASD-HL group produced more coordinate clauses than the rest of the groups (*p*s < 0.05), while both groups with ASD tended to produce fewer subordinate clauses than the TD group (*p*s < 0.05). The syntactic complexity of the narratives of ASD-LL children was lower than TD children (*p* < 0.05), while the subordination index of both groups with ASD was lower than the TD group (*p*s < 0.05).

Regarding the types of the complementizers used across the three groups, there was a significant group effect for *oti*-(that)/*pu*-(that-factive) complementizer (χ^2^ = 7.32, *p* < 0.005), stemming from the lower production of these complementizers by the ASD-LL group compared to the other two groups (*p*s < 0.001). Finally, chi-square analyses showed that the groups differed significantly on both verb-complement and modifier clauses (χ^2^ > 25.0, *p*s < 0.05). Further chi-square analyses revealed that the ASD-LL group produced significantly fewer verb-complement clauses than both ASD-HL and TD children (*p*s < 0.05). In modifier clauses, both groups with ASD produced fewer modifier, i.e., adverbial and relative, clauses than TD children (*p*s < 0.05).

### Group comparisons: macrostructural variables

Table 3 displays the total counts for the macrostructural variables, i.e., +ToM-related ISTs, -ToM-related ISTs, and story structure complexity.

**Table 3:** Group means (and *SD*s) of total counts for macrostructural measures.

**Macrostructural measure**	**ENNI (A3) story**	**ASD-HL (*N* = 16)**	**ASD-LL (*N* = 14)**	**TD (*N* = 15)**	**Chi-square (χ^2^)-value**	**Bayesian statistics (BF)**	Interpretation ^*^p < 0.05, ^**^p < 0.005, ^***^p < 0.001
+ToM-related ISTs	16 (36.4%)	4.3 (*1.7*)	3.4 (*4.4*)	4.3 (*1.6*)	8.87	5.186	ASD-LL < ASD-HL, TD^*^
–ToM-related ISTs	13 (29.5%)	2.9 (*1.6*)	1.4 (*1.5*)	5.5 (*1.5*)	10.22	8.896	ASD-LL < ASD-HL^*^ ASD-LL < TD^**^ ASD-HL < TD^**^
Story Structure Complexity (max. score: 15)	15 (34.1%)	4.8 (*2.5*)	4.8 (*2.4*)	8.4 (*0.7*)	18.79	7.047	ASD-HL, ASD-LL < TD^**^

*ASD-HL, high-language children with Autism Spectrum Disorder; ASD-LL, low-language children with Autism Spectrum Disorder; TD, typically-developing children; ENNI, Edmonton Narrative Norms Instrument (Schneider et al., 2005)*.

χ^2^ analyses showed that the groups differed significantly on the number of both +ToM-related and -ToM-related ISTs (χ^2^ > 8.87, *p*s < 0.05), as well as on story structure complexity (χ^2^ = 18.79, *p*s < 0.001). Further chi-square analyses revealed that the ASD-LL children produced fewer +ToM-related and −ToM-related terms than the rest of the groups (*p*s < 0.05), and that ASD-HL children produced fewer −ToM-related terms than TD children (*p* < 0.001). Finally, both groups with ASD scored lower than the TD group in story structure complexity (*p*s < 0.001).

### Comparisons between group scores and the original story

Table 4 presents the percentages of children in each group (TD, ASD-HL, ASD-LL) that have scored lower than the original story's baseline rates of microstructural and macrostructural measures (see Tables 2, 3). The overwhelming majority of the children in each group (>75% in each group) tended to score lower in lexical diversity and subordination, as well as in the use of *na*- (subjunctive) clauses and –ToM-related ISTs compared to the original story. Both groups with ASD (>69%), and especially ASD-LL children (86%), tended to produce fewer modifier clauses than the number of modifier clauses of the story. On the other hand, it was only ASD-LL children (>85.7%) that tended to produce stories with lower syntactic complexity, fewer verb-complement and *oti*-(that)/*pu*-(that-factive) complement clauses, as well as fewer +ToM-related ISTs than the baseline rates of the corresponding measures in the ENNI story.

**Table 4:** Percentages of children per group that scored lower than the ENNI story's baseline rates of microstructural and macrostructural measures.

**Narrative measures**	**ASD-HL (*N* = 16) (%)**	**ASD-LL (*N* = 14) (%)**	**TD (*N* = 15) (%)**
Lexical diversity	75.0	85.5	80.0
Syntactic complexity	50.0	85.7	13.3
Subordination	100.0	100.0	100.0
Verb-complement clauses	68.6	93.0	66.7
Modifier clauses	69.0	86.0	20.0
na-clauses	93.8	86,0	100.0
oti/pu-clauses	12.5	85.7	6.7
+ToM-related ISTs	69.0	100,0	60.0
–ToM-related ISTs	100.0	100.0	80.0

*ASD-HL, high-language children with Autism Spectrum Disorder; ASD-LL, low-language children with Autism Spectrum Disorder; TD, typically-developing children*.

Table 5 presents the percentages of children in each group (TD, ASD-HL, ASD-LL) that have scored higher than the original story's baseline rates in simple and coordinate clauses (see Table 2). Almost half of the children in the ASD-HL and ASD-LL group tended to produce more simple clauses than the original story, while all three groups (>93.3%) produced more instances of coordination than the number of coordinate clauses included in the ENNI story.

**Table 5:** Percentages of children per group that scored higher than the ENNI story's baseline rates of microstructural measures.

**Narrative measures**	**ASD-HL (*N* = 16) (%)**	**ASD-LL (*N* = 14) (%)**	**TD (*N* = 15) (%)**
Simple clauses	62.5	50.0	13.3
Coordinate clauses	100.0	100.0	93.3

*ASD-HL, high-language children with Autism Spectrum Disorder; ASD-LL, low-language children with Autism Spectrum Disorder; TD, typically-developing children*.

### Correlations between microstructural and macrostructural variables

Table 6 displays the results of the partial correlation analyses that focused on the exploration of possible associations between children's scores in microstructure (i.e., syntactic complexity, simple, complex, coordinate, and subordinate clauses, and types of subordinate clauses, i.e., complement and modifier), and their scores in macrostructure (i.e., +ToM-related, −ToM-related ISTs, story structure complexity), while controlling for verbal IQ and expressive vocabulary.

**Table 6:** Partial correlations between microstructural and macrostructural variables.

**Macrostructural variables**	**Microstructural variables**						
	**Syntactic complexity**	**Simple clauses**	**Complex clauses**	**Coordinate clauses**	**Subordinate clauses**	**Verb-complement**	**Modifier**
+**ToM-related ISTs**	0.50^***^	n.s.	0.49^***^	n.s.	0.66^***^	0.30^*^	n.s.
−**ToM-related ISTs**	n.s.	n.s.	n.s.	n.s.	n.s.	0.40^*^	n.s.
**Story structure complexity**	n.s.	−0.33^*^	n.s.	−0.30^*^	0.35^*^	n.s.	0.61^*^

*N = 45*.

* *p < 0.05*,

*** *p < 0.001*.

The results of the partial correlation analyses showed that the use of +ToM-related ISTs was associated with syntactic complexity, complex, subordinate, and complement clauses. On the other hand, the use of −ToM-related ISTs was only associated with the use of complement clauses. Story structure complexity was found to be positively correlated with the use of subordinate and modifier clauses, i.e., adverbials and relatives, while it was inversely associated with the use of simple and coordinate clauses.

## Discussion

In this study, we investigated the narrative retelling skills in children with ASD of high- and low-language abilities. In line with previous narrative studies (e.g., Solomon, 2004; Eigsti et al., 2007; Manolitsi and Botting, 2011; King et al., 2013; Terzi et al., 2014), we manipulated two distinct layers in narrative production: (i) microstructure, that is the intra-sentential level of narratives comprising the word or sentence complexity level of production and the relationships of the elements within sentences (Cherney, 1998), and (ii) macrostructure, which refers to the global supra-sentential level of discourse and the links among event representations that the narrator has to establish in order to build up a coherent story (Cherney et al., 1998). In the present study, we used independent language ability measures to group the children with ASD into two discrete subgroups, namely, high- and low-language ability falling in the higher and lower end of the normal range of language ability, respectively. Subsampling within the children with ASD in terms of their VIQ and expressive vocabulary scores provided the opportunity to investigate the extent to which language ability in autism is related to the children's syntactic complexity in their narratives. This procedure also allowed us to explore whether high language ability in children with ASD can boost their ability to form subordinate clauses and to attribute mental states to the story's characters. Finally, subsampling within the children with ASD enabled us to examine the role of language ability in the successful encoding of story structure and of relational information between events and characters in the story.

The data demonstrate that syntactic complexity measured in terms of the frequency of use of coordinate clauses is linked to language ability in autism. Relative to the TD group, the ASD-LL group showed significantly lower syntactic complexity, i.e., lower rates of coordinate and subordinate clauses, while there was no difference between TD and ASD-HL children on the same measure. Crucially, the ASD-LL children were the only group whose scores in syntactic complexity showed considerable deviation from the syntactic complexity pattern established in the original story, consistent with the hypothesis that ASD children's low language ability had a detrimental effect on the syntactic complexity of their narratives. A number of narrative studies report that children with ASD use a more restricted range of complex syntactic structures (Stirling et al., 2017) or less complex morpho-syntax (Tager-Flusberg, 1995; Eigsti et al., 2007; Marinis et al., 2013) in their (oral and written) narratives relative to TD children. While the children with ASD and high language abilities in the present study did not differ from their TD peers on the syntactic complexity measure, precisely the opposite obtained for the subordination index; the analyses of subordination along with the comparisons with the original story revealed that both groups with ASD tended to produce significantly fewer subordinate clauses than the TD group. As such, the analyses conducted separately for coordination and subordination provide a nuanced picture of the complexity of the narratives of the two groups of children with ASD, since the difference between ASD-HL and ASD-LL children in syntactic complexity appears to be attributed to ASD-HL children's higher coordination rather than subordination use, relative to their ASD-LL peers. Furthermore, about half of the children in each group with ASD exhibited more frequent use of simple and coordinate clauses to establish reference to the events of the story relative to the frequency pattern of simple clauses established in the original story. These structural differences between ASD and TD children may reflect a general strategy in autism to retell the story through linear, coordinated (vs. hierarchical) chains of successive events in order to safely communicate the core event structure of the story (see also Marinis et al., 2013 for similar findings).

Lexical diversity was a relative strength for both groups with ASD, since neither differed from the TD group in this narrative measure. This pattern contrasts with ASD-LL children's expressive vocabulary score which was significantly lower relative to both ASD-HL and TD children. The fact that the expressive vocabulary score of ASD-LL children did not align with their performance on lexical diversity is not surprising, given that the requirements on word use in each task are different, hence they may draw on different resources and processing constraints. In particular, word-finding in object naming is not supported by context and as such, lexical access may be more demanding than in the retelling context in which children could either recall lexical information from the story they had just listened to or rely on recalling the episodes and the context in which words were embedded in the story. Interestingly, these results are in line with Kambanaros and van Steenbrugge (2013) study on the lexical retrieval abilities of children with SLI which shows that picture naming performance was a weak predictor of the children's retrieval abilities for nouns in connected speech. Other studies also call into question the relative strength of expressive vocabulary over lexical diversity as a method for describing the lexical characteristics of the language production of children with disorders (Silverman and Bernstein Ratner, 2002; Moyle et al., 2007). However, we would like to entertain another alternative explanation for the discrepancy observed between one-word expressive vocabulary and lexical diversity in the performance of ASD-LL children. Looking into the errors that ASD-LL children made in the standardized expressive vocabulary task we notice that the low mean score did not result from “no response” data. In fact, “no response” was the least frequent type of error found. Instead, ASD-LL children tended to produce more semantic errors on average than their TD and ASD-HL peers. For instance, the ASD-LL group tended to produce semantically-related words (e.g., “spy” instead of “binoculars,” “snow” instead of “igloo”), which indicates dysfunctional lexical access rather than a limited total conceptual vocabulary.

Group comparisons on the different types of complementizers further highlight the effect of language ability on ASD children's syntactic options at the microstructural level. The ASD-LL children tended to use *oti*-(that)/*pu*-(that-factive) complement clauses at a significantly lower rate relative to the rest of the experimental groups; in fact, ASD-LL children's rates of *oti*-(that)/*pu*-(that-factive) complements substantially differed from the rates of occurrence of these complementizers in the ENNI story, with more than 80% of ASD-LL children producing fewer *oti*-(that)/*pu*-(that-factive) complement clauses compared to the input story.

We argue that this pattern of performance is due to ASD-LL children's difficulty with coordinating syntactic and lexical information being encoded in *oti*-(that)/*pu*-(that-factive) complement clauses: apart from being specified for Tense, these types of complement clauses include verbs with full specification for Aspect. An important issue in typical language development concerns the timing of acquisition of aspectual distinctions on verbs (Tsimpli et al., 2010; Kaltsa, 2012; Konstantzou et al., 2013), with relevant evidence showing that children converge on consistent, adult-like aspectual verb markings quite late mainly due to the fact that aspect marking is constrained by several syntax-semantics interface-conditioned factors, such as the aspectual class of the verb, morphological aspect and argument structure, i.e., the presence/absence of object and aspectual adverbials. Thus, aspectual distinctions must be construed from the context, presumably incurring more computational cost. We suggest that ASD-LL children may have been able to employ morpho-syntactic information to encode Tense features on the verbs of *oti*-(that)/*pu*-(that-factive) complement clauses, yet, the processing load for encoding Aspect was higher, thus resulting in lower use of the specific types of complement clauses (see Zhou et al., 2015 for similar findings). On the other hand, the less demanding morphosyntactic specification requirements of subjunctive (*na*-) clauses may account for the lack of group differences in production patterns for subjunctive complement clauses in which the options of tense and aspect verb forms are limited. Thus, both groups with ASD irrespective of language ability should be able to produce them with less strain on computational resources for language production. Nevertheless, the fact that all three groups tended to produce significantly fewer subjunctive (*na*-) clauses than those included in the original story may indicate that factors other than linguistic ones may be involved such as the depiction of the events of the story. Future work is required to incorporate the link between the use of picture-based narratives and type of subordination used in children's syntactic choices.

In addition to examining the effects of language ability on measures of syntactic complexity in microstructure, the present study has investigated the compensatory role of ASD children's language ability on the production of different types of subordinate clauses. According to our results, modifier, i.e., adverbial and relative, clauses were considerably fewer in both groups with ASD relative to TD peers. We argue that the difference between TD children and both groups with ASD irrespective of language ability indicates that morphosyntactic skills are necessary but not sufficient for the production of modifier clauses as these clauses presuppose the ability to encode coherence relations in story structure. In this respect, complement clauses are similar to modifier clauses on lexical and morphosyntactic grounds, but modifier clauses, unlike complement clauses, additionally require good discourse management skills. Interestingly, evidence in favor of modifier clauses being at the interface of syntax with pragmatics is offered by the correlation analyses of the present study. Modifer clauses are significantly positively correlated with children's scores in story structure complexity, a macrostructural measure reflecting children's ability to organize the events of the story into a pragmatically coherent whole. Overall, our findings on subordination suggest that high language ability in autism is insufficient to compensate for the production of modifier clauses whose production critically lies at the interface between syntax and pragmatics.

ASD-LL children's use of subordinate clause types in the present study affords us an opportunity to track possible similarities and differences between the retelling data of this study and Mastropavlou and Tsimpli's (2011) study with spontaneous speech data by Greek-speaking children with SLI. Both studies show that the rates of *na*-clauses were considerably higher than modifier clauses, thus, demonstrating potential overlap between the computational processes required for the production of *na*-clauses in SLI and ASD-LL children. The frequent omissions of *na* in Mastropavlou and Tsimpli (2011) in contrast to the present study where omission of complementizers was rare, can be taken as proof of severe language impairment in SLI vs. the ASD-LL children of the present study. Further similarities between the two studies may be traced in the use of *oti*-(that)/*pu*-(that-factive) complement clauses which were produced at considerably lower rates by children with SLI in Mastropavlou and Tsimpli's (2011) study in comparison to their age- and language-matched TD peers. Though the data is not directly comparable due to the different design and the age of the children recruited by the two studies, factivity seems to have caused high semantic or pragmatic (integration) costs for both ASD-LL and SLI children hence the low production of *that*-factive complement clauses. These results are consistent with our hypothesis that different types of subordinate clauses inflict distinct processing costs to children with ASD and that their computation is crucially linked to the children's language ability.

Considering macrostructure, our results show that +ToM and −ToM-related ISTs patterned differently across the two groups of children with ASD. High language ability was found to boost both IST-types, yet, more so for +ToM ISTs, bridging the distance between ASD-HL and TD children. Indeed, ASD-HL (along with TD) children were considerably more likely to produce units of information that involved characters' thoughts and feelings, i.e., +ToM-related terms, relative to ASD-LL children. Similar evidence was obtained from the comparison with the original story, since all the ASD-LL children failed to reach the +ToM-related IST frequency pattern established in the ENNI story. Crucially, the use of +ToM-related terms was found to be positively correlated with the children's rates of complement clause use. On the other hand, both groups of children with ASD were found to score significantly lower than their TD peers in the use of −ToM-related ISTs, though the ASD-HL group tended to score higher than ASD-LL children in the specific category. The discrepancy observed between +ToM and −ToM-related ISTs in children with ASD suggests that the compensatory effect of language in the domain of mental state attribution was IST-specific. As +ToM-related ISTs are prototypically used to describe mental states, i.e., the internal feelings and thoughts of others, children with autism need to recruit advanced linguistic knowledge to gain access to others' mentalistic behavior (Frith et al., 1994; Tager-Flusberg, 2000). This suggests that high language ability in autism boosts children's ability to use mental state terms predominantly when such states are relevant to the characters' feelings and thoughts.

Finally, language ability did not appear to be of critical importance to ASD children's performance in story structure complexity. The performance of both groups with ASD in story structure was equally low (i.e., 4.8 out of 15 points) and fell far below that of their TD peers. We suggest that building up the structure of a story in retelling, i.e., re-computing the story's discourse model including the setting, characters, events and outcomes, as well as the perspectives and motivations of the main characters of the story, indexed a highly demanding process of pragmatic enrichment triggered by context. Given that difficulties with pragmatic processing have been universally attested across individuals with ASD, irrespective of their age or level of functioning (Rapin and Dunn, 1997; Tager-Flusberg et al., 2005), we suggest that in situations of such demands the pragmatic deficit affects autistic children's story structure abilities over and above any language ability level. In other words, even high language skills cannot compensate for the pragmatic deficit evinced in story structure complexity. As such, in the comparison between the two macrostructural measures, i.e., +ToM-related ISTs and story structure complexity, story structure complexity seems not to be open to compensation from language skills, unlike +ToM-related ISTs. The higher contribution of pragmatics compared to language skills in developing complex story structure is independently corroborated from TD bilingual children who despite their lower language proficiency compared to monolingual children, produce more complete and elaborate stories in narrative retellings (Tsimpli et al., 2016).

Microstructure measures showed that low language ability mainly compromises the use of +ToM-related ISTs and syntactic complexity in the children with ASD, especially with respect to the use of *oti*-(that)/*pu*-(that-factive) clauses. These effects of low language ability, however, may not be unique to ASD as they do not primarily rely on good pragmatic skills, i.e., the area in which a deficit is expected in ASD. Thus, other deficits associated with low language ability, such as low working memory capacity may be responsible for the effects on these aspects of microstructure and macrostructure of narratives. Although we have no evidence to speak for or against this proposal, we believe that the narrative pattern exhibited by ASD-LL children may be uniquely associated with this group. More specifically, the asymmetry between high non-verbal and low verbal IQ (see Table 1) may be responsible for the pattern observed, leaving aside whatever pragmatic deficit characterizes ASD. To investigate this further we examined each ASD-LL child's performance in WISC-III (Wechsler, 1992) for scores in verbal and performance IQ scores following Crawford's single case approach (DISSOCS software; Crawford and Garthwaite, 2005). The difference between verbal and performance IQ scores for each ASD-LL child was tested for significant deviation from the verbal vs. non-verbal profile in the TD group. The difference between verbal and performance IQ score for all ASD-LL children differed significantly (*p* ≤ 0.01) beyond the corresponding difference in the TD group, suggesting an uneven quality of intellectual abilities in the ASD-LL group. Interestingly, Lincoln et al. (1998) meta-analytic review of 23 published studies focusing on the intellectual abilities of children with autism shows that a verbal IQ < performance IQ profile has been consistently found across studies, implying that a depressed verbal IQ relative to performance IQ score may be a marker of autism. Other studies also show that verbal IQ—performance IQ discrepancies in children could be used as an indication of a learning disability (e.g., Hyman et al., 2006). As such, recruiting a low-language ability TD group as controls for the ASD-LL children in the present study would still leave the group-matching issue unresolved as such a sample could include unidentifiable proportions of children with autistic traits or learning disabilities (D'Angiulli and Siegel, 2003). Starting from the robust verbal IQ - performance IQ discrepancy in the ASD-LL group, we assume that the patterns found in their narrative data, such as the impaired ability to construe the matrix and embedded *oti*-(that)/*pu*-(that-factive) clauses as a single event, must be specific to this population rather than simply an outcome of processing constraints that may be generalizable to TD children.

Summing up, the compensatory effect of language skills in autism has been found to be restricted to one measure of macrostructure that evaluates the use of +ToM-related ISTs, but not the other which refers to story structure complexity. The lack of an effect of language ability on the production of modifier clauses in the ASD groups suggests that both story structure complexity and modifier clauses heavily depend on the contribution of pragmatics in the syntax-pragmatics interface. As such, the compensatory role of language ability in children with ASD was confined to the microstructural measures of syntactic complexity and the production of *oti*-(that)/*pu*-(that-factive) clauses, both being more dependent on lexical and grammatical knowledge than discourse or pragmatics. We could then conclude that high language ability in autism need not always lead to improvement in narrative performance: pragmatic limitations cannot always be overridden by good language skills at least insofar as performance in a highly contextualized task like narrative production is concerned.

## Conclusions

Taken together, the results of the present study support two conclusions about the relationship between language ability and narrative performance in children with ASD. First, higher language skills enhance the syntactic complexity of narration in autism as evinced by ASD-HL children's higher use of coordinate, as well as *oti*-(that)/*pu*-(that-factive) complement clauses, relative to their ASD-LL peers. Second, high language ability was found to boost ASD children's use of ±ToM-related ISTs, with the benefit being greater for +ToM-related terms. On the other hand, in line with a number of findings reporting the universal impairment of pragmatic language in ASD, our results on modifier clauses and story structure complexity show that both high- and low language ability children with ASD performed equally low. This implies that the compensatory role of language ability in autism may not be operative in the production of subordinate clauses shaped by contextual and discourse considerations, in the use of ISTs not offering mentalistic insight into others' behavior (i.e., –ToM-related ISTs), nor in pragmatically high-demanding contexts, such as the encoding of story structure complexity. As such, the present study has suggested that higher language skills in autism are associated with a sub-set of syntactic and pragmatic competencies, a finding that would have gone unnoticed if the two groups within this normal linguistic range had been treated as a single group. Further cross-linguistic investigations of the narratives of children with ASD of various ages and language ability levels may shed more light on the extent to which their narrative performance is mediated by language ability factors. Also, future studies should recruit larger numbers of participants with ASD, so as to allow for comparisons across subgroups of more substantial sizes.

## Ethics statement

This study was carried out in accordance with the recommendations of the “Greek Institute of Educational Policy” with written informed consent from the children's parents. The parents of all the children that participated in the study gave written informed consent in accordance with the Declaration of Helsinki. The protocol was approved by the “Greek Institute of Educational Policy” within the context of the THALES research project (2012–2015) carried out by the Department of English Studies, Aristotle University of Thessaloniki, Greece (among other Departments that participated in the THALES project).

## Author contributions

All authors listed have made a substantial, direct and intellectual contribution to the work, and approved it for publication.

### Conflict of interest statement

The authors declare that the research was conducted in the absence of any commercial or financial relationships that could be construed as a potential conflict of interest.
