# Supplementary material for: Syntactic and Story Structure Complexity in the Narratives of High- and Low-Language Ability Children with Autism Spectrum Disorder
Source: Front Psychol. 2017 Nov 20;8:2027. doi: 10.3389/fpsyg.2017.02027 (PMC5701940; doi:10.3389/fpsyg.2017.02027)
Supplement: Supplementary file 1 [file Appendix.docx]

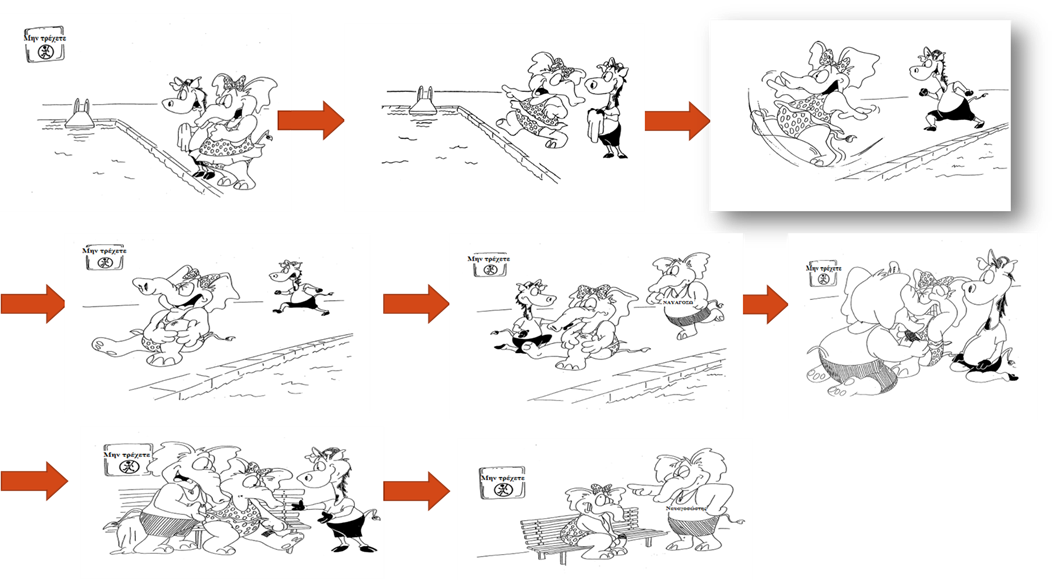
**Appendix A.** The pictures included in the A3 *Giraffe/Elephant* story of the Edmonton Narrative Norms Instrument (ENNI; Schneider et al., 2005)

**Appendix B.** The A3 *Giraffe/Elephant* story of the Edmonton Narrative Norms Instrument (ENNI; Schneider et al., 2005) that the children had to retell.

*Μια μέρα μία χαρούμενη καμηλοπάρδαλη αγοράκι, ο καμηλοπάρδαλης και μία παιχνιδιάρα ελεφαντίνα πήγαν βόλτα στην πισίνα της γειτονιάς τους. Η ελεφαντίνα αμέσως πρόσεξε μία σανίδα από την οποία μπορούσαν να κάνουν πολλές βουτιές. Κανείς τους όμως δεν είδε τη ταμπέλα που έγραφε «μην τρέχετε». Για να μη χάσουν χρόνο η ελεφαντίνα αποφάσισε να ξεκινήσει το παιχνίδι λέγοντας στον καμηλοπάρδαλη «Ας δούμε ποιος θα φτάσει πιο γρήγορα στη σανίδα!». Ξεκίνησαν το τρέξιμο με την ελεφαντίνα μπροστά και τον καμηλοπάρδαλη να την ακολουθεί. Όταν ο καμηλοπάρδαλης προσπαθούσε να τη φτάσει η ελεφαντίνα γλίστρησε και έπεσε. Ο φοβισμένος καμηλοπάρδαλης είδε ότι αυτή πονούσε και έκλαιγε. Ένας ελέφαντας ναυαγοσώστης τους είδε και πλησίασε προς το μέρος τους για να δει ποιο ήταν το πρόβλημα. Η ελεφαντίνα του εξήγησε κλαίγοντας τι συνέβη. Ο ναυαγοσώστης αφού εξέτασε την πληγή της της έβαλε ένα τσιρότο, καθώς ο ανήσυχος φίλος της παρακολουθούσε γονατιστός. Αμέσως τη βοήθησαν να περπατήσει μέχρι το παγκάκι για να ξεκουραστεί. O φίλος της ο καμηλοπάρδαλης ανακουφίστηκε και χάρηκε που η φίλη του φαίνονταν καλύτερα. Μόλις έφυγε ο φίλος της, ο ναυαγοσώστης κοίταξε την ελεφαντίνα αυστηρά και της έδειξε την πινακίδα που έγραφε «μην τρέχετε» και της είπε ότι την επόμενη φορά θα πρέπει να είναι πιο προσεχτική. Εκείνη του το υποσχέθηκε και τον ευχαρίστησε που τη βοήθησε.*

Translation in English: One day a happy giraffe boy, called Giraffo, and a playful elephant girl, called Elephantina, went out to the neighborhood pool. Elephantina noticed a diving board from which they could dive as many times as they wanted. But neither of them saw the sign which said "NO RUNNING!" Elephantina, in order not to waste time, decided to start a game by saying to Giraffo "Let's see who can reach the diving board first!". They started running with Elephantina in front and Giraffo following her. While Giraffo was trying to reach her, Elephantina slipped and fell. Scared Giraffo saw that she was in pain and she had burst into tears. A lifeguard elephant saw them and walked towards them to see what the problem was. With tears in her eyes, Elephantina explained to the lifeguard what had happened. The lifeguard, after having examined the wound, put a band-aid on it, while her concerned friend was watching while kneeling next to her. They immediately helped her to walk over to a bench to relax. Her friend, Giraffo, was relieved and glad that his friend looked well. As soon as her friend left, the lifeguard looked strictly at Elephantina and pointed to the sign that said "NO RUNNING!" and told her that she should be more careful next time. She promised to do that and thanked him for helping her.

**Appendix C.** Examples of the story structure of the narratives (in *italics*) of a TD, an ASD-HL and an ASD-LL child (A3 *Giraffe/Elephant* story; the Edmonton Narrative Norms Instrument) (ENNI; Schneider et al., 2005).

1. LK (ASD with high language ability)

*Μια φορά κι έναν καιρό μια μέρα η ελεφαντίνα και η καμηλοπάρδαλη πήγαν στην πισίνα.*

[Once upon a time one day the elephant-girl and the giraffe went to the swimming pool]

*Η ελεφαντίνα είδε κάτι που πήγαμε και κάνουμε βουτιές.*

[The elephant saw something where we went and dive].

*Κανείς όμως δεν παρατήρησε ότι υπήρχε μια πινακίδα που έλεγε “Μην τρέχετε”.*

[Yet, nobody observed that there was a sign which said "NO RUNNING!"]

*Και η ελεφαντίνα και η καμηλοπάρδαλη έκαναν αγώνες για το ποιος θα φτάσει εκεί.* [And the elephant-girl and the giraffe raced to see who will reach it first]

*Όμως προσπαθώντας η καμηλοπάρδαλη να προσπεράσει την ελεφαντίνα η ελεφαντίνα γλίστρησε κι έκλαιγε.*

[But while the giraffe was trying to bypass the elephant-girl, the elephant-girl slipped and cried]

*Μετά ήρθε ο ναυαγοσώστης, της είπε να πει τι έγινε.*

[Then the lifeguard came, he told her to say what happened]

*Ο ναυαγοσώστης είπε στην ελεφαντίνα να καθίσει στο παγκάκι.*

[The lifeguard told the elephant-girl to sit on the bench]

*Η καμηλοπάρδαλη ανακουφίστηκε που η ελεφαντίνα ήταν μια χαρά.*

[The giraffe felt relieved that the elephant-girl was fine]

*Όταν έφυγε, ο ναυαγοσώστης πήρε αυστηρό ύφος και της έδειξε την ταμπέλα που λέει ‘Μην τρέχετε’ και από τότε δεν έτρεξε ποτέ.*

[When (he) left, the lifeguard looked strictly and pointed the sign to her that says “NO RUNNING!" and since then she never ran]

*Και ζήσαμε αυτοί καλά κι εμείς καλύτερα.*

[And we lived happily ever after]

1. GB (ASD boy with low language ability)

*Το κορίτσι ελέφαντας χτύπησε και έπεσε κάτω.*

[The elephant-girl hit and fell down]

*Το κορίτσι είχε σπάσει το πόδι του κι ο πατέρας της τη διόρθωνε.*

[The girl had broken her leg and her father fixed her]

*Η ελεφαντίνα ήθελε να πάει πρώτη εκεί στην πισίνα αλλά η καμηλοπάρδαλη αυτή ήθελε πρώτη.*

[The elephant-girl wanted to go there to the swimming pool first but the giraffe, she wanted (to go) first]

*Όμως η ελεφαντίνα γλίστρησε και χτύπησε το πόδι της.*

[But the elephant-girl slipped and hit her leg]

*Μετά ήρθε ο πατέρας της και της έβαλε γάζα.*

[Then her father came and put a band-aid on her]

*Ο πατέρας της είπε ότι θα είναι καλά κι ο μπαμπάς-ελέφαντας έδιωξε την καμηλοπάρδαλη.*

[Her father said that (she) will be fine and the father-elephant sent the giraffe away]

1. GP (typically-developing boy)

*Μια μέρα συναντήθηκαν σε μια μεγάλη πισίνα δύο φίλοι*

[One day two friends met at a big swimming pool]

*ένα κοριτσάκι ελέφαντας και ένα αγοράκι καμηλοπάρδαλη.*

[an elephant-girl and a giraffe-boy]

*Έπαιζαν διάφορα παιχνίδια, έτρεχαν από δω και από εκεί,*

[They were playing several games, they were running around]

*γιατί είχαν πολύ καιρό να πάνε στην πισίνα*

[because it’s been a long time since the last time they went to the swimming pool]

*και ήθελαν να κάνουν όσα περισσότερα πράγματα προλαβαίνουν.*

[and they wanted to do as many things as they could]

*Τότε η ελεφαντίνα αφού είδε ότι υπάρχει ένας βατήρας δίπλα τους,*

Then the elephant girl, since she saw that there is a diving board near them]

*είπε στον φίλο της: Θες να κάνουμε έναν αγώνα για να δούμε ποιος θα φτάσει πρώτος?*

[said to her friend: Do you want to race to see who will reach (the diving board) first?]

*Άρχισαν να τρέχουν όμως η ελεφαντίνα έτρεξε πολύ γρήγορα και έπεσε και χτύπησε*

[They started running but the elephant-girl ran very fast and she fell and hit]

*Το αγοράκι καμηλοπάρδαλη στενοχωρήθηκε πάρα πολύ*

[The giraffe-boy was very sad]

*γιατί η αγαπημένη του φίλη χτύπησε.*

[because his loving friend hit]

*Για καλή τους τύχη εκεί κοντά ήταν ένας ελέφαντας ναυαγοσώστης*

[Fortunately there was an elephant lifeguard nearby]

*που είδε τι έγινε*

[who saw what happened]

*και θέλησε να βοηθήσει.*

[and (he) wanted to help]

*Όταν έφτασε εκεί είδε την ελεφαντίνα που έκλαιγε*

[When he arrived there, he saw the elephant-girl crying]

*και την ρώτησε τι συνέβη.*

[and asked her what happened]

*Της είπε ότι δεν χρειάζεται να κλαίει γιατί αυτός θα μπορούσε να την βοηθήσει.*

[He told her that there is no reason to cry because he could help her]

*Της έδεσε το πόδι για να μην τρέχει το αίμα*

[He tied her leg so that it won’t bleed]

*και της είπε ότι την επόμενη φορά θα πρέπει να είναι πιο προσεχτική*

and told her that next time she needs to be more careful]

*γιατί υπάρχει πινακίδα*

[because there is a sign]

*που γράφει ότι δεν πρέπει να τρέχουν τα παιδάκια.*

[that writes that children must not run]
